# From means and variances to persons and patterns

**DOI:** 10.3389/fpsyg.2015.01007

**Published:** 2015-07-24

**Authors:** James W. Grice

**Affiliations:** Department of Psychology, Oklahoma State University, Stillwater, OK, USA

**Keywords:** observation oriented modeling, integrated model, inference to best explanation, mean, variable-based modeling

## Abstract

A novel approach for conceptualizing and analyzing data from psychological studies is presented and discussed. This approach is centered on model building in an effort to explicate the structures and processes believed to generate a set of observations. These models therefore go beyond the variable-based, path models in use today which are limiting with regard to the types of inferences psychologists can draw from their research. In terms of analysis, the newer approach replaces traditional aggregate statistics such as means, variances, and covariances with methods of pattern detection and analysis. While these methods are person-centered and do not require parametric assumptions, they are both demanding and rigorous. They also provide psychologists with the information needed to draw the primary inference they often wish to make from their research; namely, the *inference to best explanation*.

## Introduction

In his erudite and now classic book *Constructing the Subject*, Danziger (1990) describes how psychology came to be dominated by an approach toward data conceptualizing and analysis he dubbed the “triumph of the aggregate.” Charting the meteoric rise of tables of means, variances, correlations, and other aggregate statistics reported in psychology journals from the early to mid-1900s, Danziger (1990) lamented the corresponding demise of the individual subject, or person, in psychology. Aggregate statistics were moreover shown to rise in prominence despite cautionary claims regarding their hegemony (Sidman, 1952; Bakan, 1954), including a critical appraisal offered by none other than Skinner (1956) himself. Modern scholars point out the issues raised over 50 years ago have not gone away and that, in fact, psychology’s over-reliance on aggregate statistics is likely thwarting scientific progress by hindering the development of theories which can explain the behavior of individual persons (Valsiner, 1986; Valsiner et al., 2014). Lamiell (1997, 2003, 2013) has gone to great lengths to remind personality psychologists, in particular, that between-person differences or effects discovered through aggregate statistical analysis do not necessarily exist at the level of the individual (see also, Carlson, 1971). The Big Five personality factors, for example, can readily be found in aggregated data, but the factors do not regularly emerge from the analysis of individual responses (Grice et al., 2006; see also, Molenaar and Campbell, 2009). The Power Law of Learning is another example phenomenon that can be seen in the aggregate but not at the level of the individual, thus raising the question of whether or not it is truly a law (see Heathcote et al., 2000, as reported by Speelman and McGann, 2013). There is a genuine and potentially hazardous disconnect, then, between conclusions drawn from between-person, aggregate statistics and statements or theories meant to offer insight into the psychology of individual persons.

In this paper we present a framework for conceptualizing and analyzing data that does not rely on traditional aggregate statistics such as the mean, median, variance, covariation, etc. Instead, this approach—like Exploratory Data Analysis (EDA; Tukey, 1977; Behrens and Yu, 2003)—relies primarily upon techniques of visual examination to detect and explain dominant patterns within a set of observations. Going beyond EDA, however, this approach can incorporate patterns that are generated *a priori* on the basis of theory, thus promoting model building and development. It also synchronizes visual examination of the data with transparent analyses that (1) identify those individuals whose observations are consistent with the predicted or identified pattern, and (2) provide an index of a given pattern’s robustness within a sample. This approach is generally referred to as *observation oriented modeling* (OOM; Grice, 2011, 2014), and we will compare and contrast its guiding principles and techniques with those of traditional statistics using a study and accompanying data that are contrived but nonetheless based on genuine psychological research. We will also draw, in part, upon Haig’s (2005; 2014) *abductive theory of method* (ATOM) to argue that this approach provides the types of inferences psychologists normally seek from their data but are unable to make on the basis of traditional statistical analyses. The overall goal is to show that by departing from the modal research practice of modern psychology (Lebel and Peters, 2011), a novel and more rigorous path that does not confuse aggregates for persons may be paved for future researchers.

## An Example Study in Rejection

Pick up almost any research paper on human psychology, and there you will find written in the Introduction statements about persons. You will not likely find statements about means, variances, or even covariances; although you might find descriptions of relationships between different attributes or qualities. Even these relationships, however, will be discussed in the context of living persons rather than aggregate statistics. Writing about rejection and interpersonal coping, for example, Ayduk et al. (2003) claim “…research suggests that people who fear and expect rejection employ to a greater degree both overt (i.e., verbal aggression) and covert (i.e., withdrawal, avoidance) negative coping strategies that ultimately undermine their significant relationships and their mental health” (p. 435). Here rejection and interpersonal coping are foremost recognized as universal features of human experience. It is indeed difficult to imagine any adult who could not recall an instance of being rejected by another person or recount a situation in life that was coped with in a negative, unfruitful manner. The authors moreover infer from previous research—naturally based on a limited number of individuals—that rejection and coping are causally connected. The inference is therefore from samples of persons to persons in general, as is clear with the authors’ use of the word “people.” Concomitant with this inference is the conclusion that rejection and coping are causally connected, not at the aggregate or even group level, but at the level of the person. For any given individual, then, the chronic expectation of rejection (likely developed from a history of being rejected by others) plays a causal role in the generation of negative coping strategies. How did the authors draw such inferences, and are these types of inferences truly warranted when made on the basis of results obtained from traditional, aggregate statistics? The answer to the second question is “no,” and to understand how psychologists typically draw such conclusions from their analyses, we must work patiently and carefully through an example study.

Continuing with the topic at hand, rejection can be produced and studied in the laboratory by psychologists (e.g., Downey and Feldman, 1996; Ayduk et al., 2003). Consider, for instance, a male college student (viz., “the participant”) who walks into a laboratory and is informed that he will be interacting, via the Internet, with another male student seated at another computer across campus. The participant is asked to provide a short biography to share and is then given a corresponding biography from his counterpart on the other side of campus. The biography presents a person who is kind and inquisitive and likely a pleasure to interact with in an informal social setting. After the participant reads the biography and prepares for the online interaction, the experimenter receives a phone call and informs the participant that his counterpart has now chosen not to participate in the online discussion and is instead withdrawing from the study. What is the participant to make of this unexpected decision? The experimenter’s hope is that the student will in fact interpret the counterpart’s decision as a rejection of the participant based on his shared biography. The experimenter moreover expects the participant to subsequently experience negative emotions, make negative self-attributions, and to form a negative inclination toward his rejecting counterpart. With the phone call and rejection completed, the experimenter then asks the participant to judge the counterpart on qualities such as intelligence, popularity, and friendliness using a 6-point rating scale. These ratings essentially provide the student with an opportunity to express his displeasure with the rejecting partner. After making his ratings, the participant is finally debriefed and informed of the deception; viz., no other student was involved in the study, and the biography and ostensible rejection were therefore not genuine.

**FIGURE 1:**
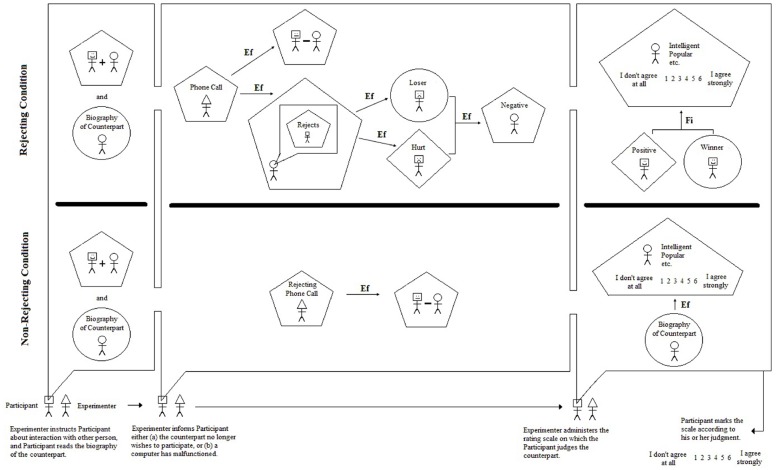
**Integrated model for rejection study**.

Now imagine over the course of a semester eighty individual students walking into the psychologist’s laboratory and being guided through these same procedures. With each and every student the experimenter’s expectations will be the same, because within her mind is a model. Perhaps it is a model that is only crudely elaborated, but it is a model nonetheless that is meant to explain the thoughts, feelings, and behaviors of each individual student (person) in the study. What might this model look like? The most rigorous way to express the model is via a picture like that shown in Figure 1. Such pictures are referred to as *iconic* or *integrated* models because they provide a visual snapshot of the structures and processes, or causes and effects, at work in the laboratory (i.e., at work in the participant, experimenter, and setting) during the study. As can be seen on the left side of Figure 1, the model depicts two conditions in the study. The top part of the model, demarcated by the bold line, represents what takes place in the study as described in detail above. The bottom part of the model will be described later.

The model also demarcates three important points of interaction between the experimenter and participant. The purpose of the first interaction, from the perspective of the experimenter, is to create within the mind of the participant an expectation of an online interaction with another student across campus. The pentagon enclosing the image of the participant and counterpart joined by a “+” sign represents a simple or complex judgment. In this instance, the participant judges that he will be interacting with the other student, and that the interaction will be positive (given the biography and the experimenter’s instructions). The circle enclosing the image of the counterpart represents certain predicates (predicative adjectives, predicative nouns) based on the biography. For example, the biography describes the counterpart as “a student,” “a psychology major,” “outgoing,” etc. The counterpart student is thus known through the neutral and positive descriptive nouns and adjectives given in the biography.

The purpose of the second interaction (focusing on rejecting the condition) is to inform the participant that the other student has chosen not to participate in the discussion after having read the participant’s biographical statement. It is not perfectly clear or stated plainly, however, if the counterpart is rejecting the participant, but it is the experimenter’s expectation that the participant will interpret this decision as a personal rejection based on his own biographical statement. The phone call is therefore considered to be an efficient cause; that is, a cause that proceeds its effect in time leading to its production or change (denoted by an arrow labeled “Ef” in the model; see Grice, 2014). The resulting judgment of the counterpart rejecting the participant is represented by the pentagon in the second interaction of the study. It is also accompanied by a second effect of the phone call; specifically, the simple judgment that the two will not communicate via the Internet after all.

The judged rejection then operates as an efficient cause of negative self-predications (negative self-attributions) by the participant. These negative self-predications are represented by the circle derogatorily labeled “Loser” enclosing the participant in the second interaction of the study. Hurt feelings, represented by a diamond labeled “Hurt” enclosing the participant, also result from the judged rejection. Finally, these experiences occurring simultaneously within the participant cause him (as an efficient cause) to adopt a negative disposition toward the counterpart. This negative disposition may occur consciously, for instance, if the participant were to think disparaging thoughts such as “well, that guy’s a jerk for wasting my time” or “I always knew psychology majors were unstable.”

Finally, the purpose of the third interaction is to provide the participant with an opportunity to make explicit judgments about his rejecting counterpart. As can be seen on the right side of Figure 1, the participant rates his counterpart using a 6-point scale anchored by “I don’t agree at all” and “I agree strongly.” The participant is asked to use the scale for nine adjectives that may describe the counterpart (intelligent, popular, friendly, etc.). Use of the scale is considered to be a complex judgment task as indicated by its enclosure in a pentagon. What value, or potential range of values, should the participant choose? At this point, the model is not sufficiently developed to specify exactly which values will be chosen, but it does explicate the most proximate cause behind the selection. Specifically, the participant will choose a value that will result in positive feelings and positive self-attributions, as indicated by the diamond labeled “Positive” and the circle labeled “Winner” in the figure. He is therefore attempting to reach a goal through his rating, and goals operate as final causes in human behavior (see Rychlak, 1988; Grice, 2014). The arrow therefore points from the positive feelings and positive self-attributions and is labeled “Fi” for “final cause.” Here the participant is essentially trying to make himself feel better through the rating judgment by discounting the source of his negative feelings and negative self-attributions (viz., the counterpart). It is therefore reasonable to posit that the participant will choose one of the low values on the scale (1, 2, 3), which would ostensibly indicate negative judgments of unintelligent, unpopular, unfriendly, etc.

At the end of the model (bottom right-hand corner of Figure 1) is the only “output” the experimenter observes; namely, the circled ratings for each adjective. No other attempts are made in the study to observe the predications, judgments, or feelings of the participant. Nonetheless, the figure spells out very clearly, for everyone to see, the structures and processes thought to be at work by the experimenter when the participant is ostensibly rejected. Finally, the model also shows a second condition of the study in which the participant is told that, due to a computer malfunction, the counterpart will not be able to take part in the discussion. The participant is still asked to rate his counterpart, but as can be seen in the model, all of the important predications, judgments, feelings, and causes are no longer present. His rating is driven by the biographical sketch, as an efficient cause, remembered from the beginning of the study. In this case, it would be reasonable to posit that the participant will choose one of the high values on the scale (4, 5, 6) due to the positive content of the sketch. Again, for both the rejecting and non-rejecting conditions, however, predictions for the scale values are not explicitly provided by the model.

### Three Important Inferences

The integrated (iconic) model in Figure 1 facilitates three inferences the psychologist wishes to make through her research efforts—even if she is not consciously aware of these inferences—and they are the types of inferences described at the beginning of this paper. The first is known as an *abductive inference* (or simply *abduction*) which has its roots in Aristotelian philosophy and was developed and popularized by the American philosopher Charles S. Peirce (Haig, 2005; Flórez, 2014). In order to understand this inference, let us suppose that all of the participants in the rejecting condition selected “I don’t agree at all” for each and every adjective as applied to the counterpart. The data obtained from the study are recorded as whole numbers valued 1 through 6, and here all of the observed values for the rejected participants are 1’s. Why do the numbers show this striking pattern? The experimenter’s answer to this question, that is her conclusion or inference, is that the data are patterned in this manner because of the structures and processes detailed in the model. The specific form of this abductive inference can be represented as follows:

1’s have been observed for the rejected participants.

If the structured processes in Figure 1 took place, 1’s would have been observed.

_________________________________________________________________

Therefore, the structured processes in Figure 1 took place.

A key feature of the inference is that, unlike induction, it appeals to explanation (viz., the structures and processes diagrammed in Figure 1). The conclusion is also uncertain or provisional, unlike conclusions reached through strict deductive reasoning. This uncertainty rests partly upon the iconic model itself as it is not sufficiently developed to predict that only 1’s will be selected by the participants. Moreover, it is not clear if 1’s should be selected for all nine adjectives, or if 2’s and 3’s are also expected since they lie below the mid-point of the scale, ostensibly conveying a negative judgment. Indeed, it is not clear why a 6-point rating scale is being used rather than, for instance, a 5-point scale or a binary judgment task. These uncertainties are part and parcel of the inference being sought and they should not be viewed as reasons for abandoning the explanatory model. Instead, these insufficiencies should be viewed as a call to make improvements to the model in Figure 1 through refinement and extension.

The model can be refined by changing its existing components; for example, the exact emotions felt by the participant can be elaborated, or the 6-point scale can be justified and predictions included about how the participant should behave with regard to the scale. The model can be extended by adding additional components; for example, perhaps not every participant will construe the counterpart’s actions as rejection, and the determining factors for making such an interpretation can be added to the model. Of course the entire model itself can be tested against and perhaps superseded by a competing model (such as a Freudian view of hostility). In this regard, in particular, the experimenter is seeking an *inference to best explanation*, which is a type of scientifically useful abduction with the general form,

D is a collection of data

H (an hypothesis) would, if true, explain D

No other hypothesis can explain D as well as H does

_________________________________________________________________

Therefore, H is probably true

The conclusion is again uncertain, but the continual striving to thoroughly evaluate, improve upon, or replace a given model seems to capture the investigative spirit of modern science, at least as it is idealized. In any case it is easy to see why Haig (2014), in his ATOM, regards inference to best explanation as central to developing a proper understanding of science.

The third inference sought by the experimenter is an attempt to draw a conclusion about persons, in general, from her specific sample of individuals in her particular study. The model in Figure 1 is clearly tied to the study designed by the experimenter and is therefore applicable, she hopes, to each and every participant in her study. Beyond these persons, however, the components of the model provide a potential explanation for how persons, in general, might react to being rejected. The second interaction between the experimenter and participant, for instance, shows a causal link between the judgment of being rejected by another person and the negative self-attributions and feelings experienced by the participant. The third interaction moreover shows that the proximate cause of a negative, explicit judgment toward a rejecting person is a final cause resulting in positive feelings and self-attributions. Presuming the observations do in fact support the model in her study, the experimenter can then argue that these components of the model may offer valid explanations of how persons react to rejection in situations outside the laboratory. In doing so, the psychologist will be reasoning inductively, moving from the specific to the general, and it is in this way that psychologists typically seek to generalize beyond their samples of participants and particular laboratories.

The three types of inference sought by the experimenter in this example are therefore (1) abduction, (2) inference to best explanation, and (3) inductive generalization. All three inferences are facilitated by the integrated, iconic model in Figure 1; indeed, it could be argued that such models are indispensable for making these inferences. In any case the inferences are clearly important, and as noted at the beginning of this paper, they are the types of inferences encountered in the Introduction and Discussion sections of journal articles published throughout psychology. It is also important to point out that none of these inferences is tied explicitly to a mean, median, mode, variance, or any other aggregate statistic that can be computed from a sample of data. The integrated model was designed without any statistical procedure in mind and without the restriction of only including features that can be understood quantitatively. All 10 of Aristotle’s categories of being, and all four of his causes can be incorporated into an integrated model (see Grice et al., 2012; Grice, 2014). The theoretical horse, so-to-speak, is therefore in front of the data analytic cart, as it should be. In more sophisticated language, we are not letting our methods determine our metaphysics (Rychlak, 1988).

### One Underwhelming Inference

When psychologists argue they are using statistics to generalize beyond their samples, it is important to realize most believe they are generalizing in the manner described above; namely, making an abductive inference to best explanation or making an inductive inference about persons in general. Unfortunately, in the overwhelming majority of cases, nothing could be further from the truth. By using traditional statistical methods that rely on null hypothesis significance testing (NHST; viz., traditional *p*-values), psychologists are instead routinely making an *inference to a population parameter*, which is far less informative and far less useful for building scientific theories than the three inferences drawn from integrated models described above.

**FIGURE 2:**
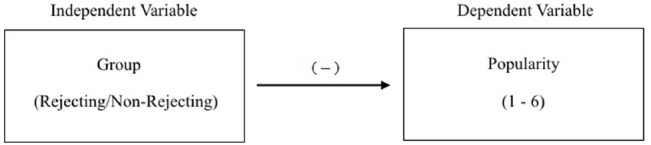
**Variable-based model for rejection study**.

To demonstrate this ubiquitous type of inference, let us now consider the condition in which the participant is told that his counterpart cannot participate in the online discussion because of a computer malfunction. With this comparison group in place, and following modal research practice (Lebel and Peters, 2011), the experimenter now thinks about the study using the variable-based model in Figure 2. As can be seen, this model is comprised of an independent variable (viz., group) and dependent variable (viz., popularity rating) connected with a line that represents their relationship or correlation. The negative sign above the line indicates that those in the rejecting condition are expected to, on average, provide lower ratings than those in the non-rejecting condition. In order to keep everything simple, we will henceforth only consider the rating for “popular” in the analyses. Given the dichotomous group membership variable and rating scale with values ranging from 1 to 6, the experimenter follows standard protocol and analyzes the data with an independent samples *t*-test. Her results, obtained from 160 participants, reveal a statistically significant difference between the rejecting (*M* = 4.20, SD = 0.40) and non-rejecting (*M* = 4.50, SD = 1.21) groups, *t*(96.23) = –2.10, *p* < 0.04, *d* = 0.33, CI_0.95_: –0.58, –0.02. The difference is also consistent with expectation, with the rejecting group yielding a lower mean than the non-rejecting group.

What inference can she draw from these results, assuming she has met or properly adjusted for all of the assumptions of the statistical test? Having used NHST, the experimenter posited two populations from which she drew her samples: a population of persons experiencing rejection in the study, and a population of persons not experiencing rejection in the study. The populations in this example, as in most studies in psychology, are entirely imaginary (Berk and Freedman, 2003); but nonetheless a mean rating value is presumed to exist for each, designated as μ_1_ and μ_2_. The null hypothesis is that the two population means are equal (H_0_: μ_1_ = μ_2_) and by declaring her results as “statistically significant” she has rejected this hypothesis and concluded (inferred) that the two population means are not equal. She can consider the difference between the population means as a parameter to be estimated as well (viz., μ_*diff*_ = μ_1_ – μ_2_) and then provide a point estimate for what she thinks the difference might be (viz., 4.20 – 4.50 = –0.30). She can also provide an interval with an assigned level of confidence for possible values of the difference (viz., CI_0.95_: –0.58, –0.02).

With the point and interval estimates in hand, it is clear the psychologist is attempting to make an inference to a population parameter (μ_diff_), which is presumably fixed at some value. This inference is the only one she can rationally make; and the term “rationally” should be used loosely here because the low observed *p*-value (*p* < 0.04) does not provide the probability of the null hypothesis being true, and therefore worthy of rejection. The *p*-value instead indicates the two-tailed probability of obtaining a *t*-value of at least 2.10, assuming the hull hypothesis is true (see Cohen, 1994). Regardless, she cannot make an abductive inference due to the simplicity and nature of the variable-based model. The model is not explanatory as it does not present the structures and processes underlying the observations. It simply conveys the mean difference between two variables for arbitrarily defined populations. According to Haig’s ATOM such a model with the accompanying parameter estimation may contribute to phenomena detection, which can play an important role in science, but the psychologist must be clear that the only conclusion she can draw from her analysis is that, provided the assumptions for the independent samples *t*-test have been met or adjusted for appropriately, the mean population difference is not 0, consistent with expectation, and is instead estimated with 95% confidence to be encompassed by values ranging from -0.02 to -0.58. That is all.

The experimenter also cannot make an inductive inference to people in general as her hypotheses and analysis are constrained to means. She cannot, therefore, write statements such as “people who are rejected will rate the rejecting person as less popular than those who are not rejected” or “rejected persons, compared to non-rejected persons, considered the counterpart to be unpopular.” In order to be true to her model and analyses, she must restrict her inferential statements to population means or the difference between them. Moreover, she must be careful to avoid the following erroneous conclusions from her statistically significant finding:

• Because my result was statistically significant, it will likely replicate across independent samples of participants.
• My result is not likely due to chance given the low *p*-value.
• The null hypothesis is probably false; that is, the probability the null hypothesis is true is less than five percent.
• My research hypothesis is probably true.

Lambdin (2011) reports a more complete list of twelve such errors commonly made by researchers in psychology, education, sociology, medicine, and other disciplines who rely on null hypothesis significance testing (i.e., common *p*-values) to determine the scientific value of their results.

## Getting Beyond Aggregate Statistics and NHST

**FIGURE 3:**
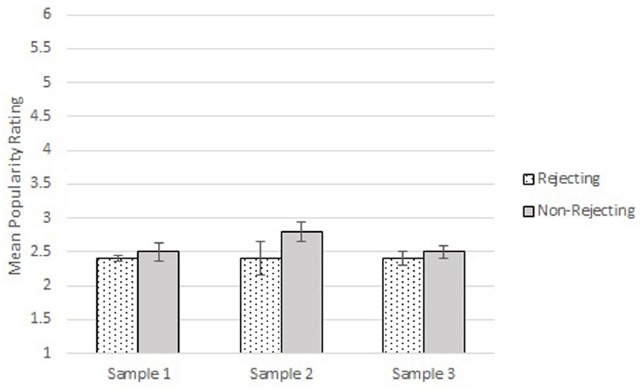
**Means and standard errors for rejecting and non-rejecting groups for each of the three samples**.

A side-by-side comparison of the models in Figures 1 and 2 shows clearly the integrated model is much more informative and rigorous than the variable-based model. The arguments above have also shown that the integrated model provides a gateway for the experimenter to make the types of inferences she truly wishes to make, whereas the variable-based model permits only a restricted, low information inference to a population parameter. In order to drive home the point that the latter inference is low in information, let us consider two additional samples of participants collected by the same experimenter using the exact same experimental protocol with a rejecting and non-rejecting condition. The descriptive statistics, *t*-values, *p*-values, and confidence intervals for all three samples are reported in Table 1, and the means and standard errors are displayed in bar graphs in Figure 3. As can be seen in the table, using these metrics she may conclude that the initial results have been replicated in the two new studies. The effect sizes, in particular, are equal (*d* = 0.33) when reported with two decimals of precision.

**TABLE 1:** **Statistics and independent samples *t*-test results for three studies**.

	**Condition**				
	**Rejecting**		**Non-rejecting**	
Sample	*M*	SD	*M*	SD	*M*_diff_	*d*	*t*	*p*	CI_0.95_
1	4.20	0.40	4.50	1.21	–0.30	0.33	–2.10	0.037	–0.58, –0.02
2	4.20	2.24	4.80	1.34	–0.60	0.33	–2.06	0.042	–1.18, –0.023
3	4.20	0.89	4.50	0.93	–0.30	0.33	–2.10	0.038	–0.58, –0.02

The t-values and p-values for Samples 1 and 2 were adjusted for violations of the homogeneity of population variances assumption. All sample sizes were equal to 80.

**FIGURE 4:**
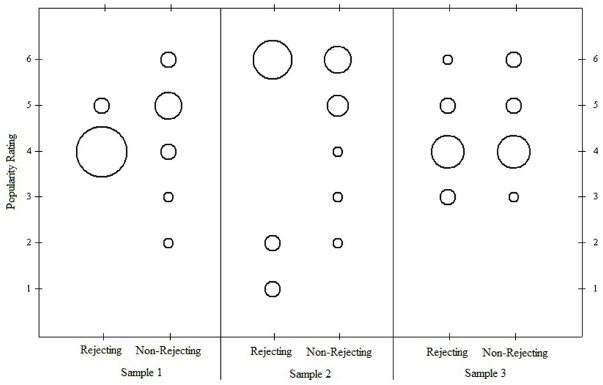
**Bubble plots for three samples of participants.** Larger bubbles indicate greater numbers of cases.

What do we really know about these data based on Table 1 and Figure 3? Simple bubble plots surprisingly indicate that important information has been overlooked by focusing only on the tabled statistics. The bubble-plot in Figure 4 for the first and original data set shows radical differences between the two groups with respect to the variability and distributions of their scores. While an overwhelming majority of participants in the rejecting group chose 4’s, participants in the non-rejecting condition chose values ranging from 2 to 6. The second bubble-plot indicates a radical divide in the distribution for the rejecting group, with participants choosing only 1’s, 2’s, or 6’s; whereas the distribution for the non-rejecting group shows skew toward the lower values on the scale. Finally, the third bubble-plot indicates the observed values are distributed similarly across the 6-point scale, with a slight tendency for participants in the rejecting group to select 3’s and a slight tendency for participants in the non-rejecting group to select 6’s.

The results from the three studies clearly show different patterns of responses that are simply not detectable in the aggregate statistics or bar charts. What is the experimenter to do? She could switch to a non-parametric procedure, but there are clear incentives for not doing so, including the potential loss of statistical power and the unwarranted perception that such a switch would indicate weakness in her methods and results. A median test in fact yields statistical significance for only the first two data sets. She could switch to a Bayesian analysis which would permit her to compare means while also assessing parameters relevant to the distributions of the samples. For all three data sets the Bayesian analysis in fact indicates “credible differences” between the group means, as the Highest Density Interval excluded 0 in each case. Fundamentally, though, none of these options represents a departure from the variable-based model in Figure 2 and the goal of estimating parameters. In other words, like the independent samples *t*-test, effect size, and confidence interval these approaches would not require nor encourage the experimenter to explicate the structures and processes at work in or outside of her laboratory regarding the human experience of rejection.

The first step toward a more rigorous analysis of the data that is also consistent with the types of inferences sought through the model in Figure 1 is to consider the detection and explanation of patterns as more generally important than parameter estimation (see Thorngate, 1986; Manicas, 2006). The experimenter has two key observations for each participant: (1) whether or not the participant was rejected, and (2) the participant’s ratings using the 6-point scale. Here, as above, we will only consider the rating for popularity, and the two observations together create a simple two-dimensional array:

**Figure:**
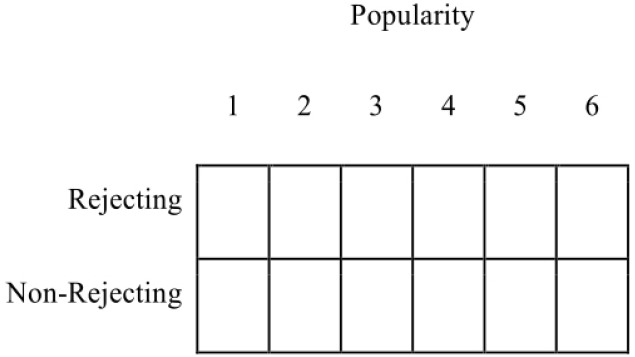


Given the experimenter’s choices, then, this array presents boundaries on the ways she thinks data can be structured, and it is within this limiting structure she must identify or search for meaningful and robust patterns of observations.

### Deductive, *a priori*, Pattern Evaluation

**FIGURE 5:**
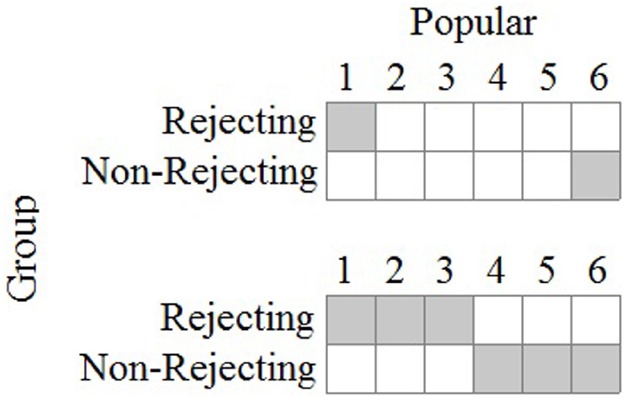
**Predicted patterns of observations for rejection study.** Gray cells indicate predicted joint observations for group membership and popularity rating.

If the model in Figure 1 were sufficiently developed, the experimenter would approach her data in a way most similar to deductive reasoning. In the parlance of modern research design and statistical analysis, she would conduct *a priori* tests of the model’s accuracy which would require specific predictions about the observations. Figure 5 shows two example predicted patterns using the two-dimensional array above that might be consistent with the integrated model. The first pattern shows that the experimenter expects participants in the rejecting group to select 1’s (“I don’t agree at all”) on the 6-point scale and participants in the non-rejecting group to select 6’s (“I agree strongly”). The second pattern shows that the rejected participants are expected to choose values 1, 2, or 3, while the non-rejected participants are expected to choose values 4, 5, or 6. These patterns are consistent with the model insofar as rejecting participants are expected to discount the counterpart, and lower values are interpreted as indicating negative judgments of low popularity.

Of course other patterns could be put forth as examples, but the point here is that if the experimenter is to work deductively and conduct *a priori* tests, she must develop the integrated model beyond what is shown in Figure 1. If she continues to employ a 6-point scale, she must be able to make predictions about which specific values will be selected by all—or at least a majority of—her participants. Such predictions will no doubt be difficult and will require extensive research into how individuals interpret and respond to the rating scale, but this is the demanding and often tedious scientific work required for accomplishing a better understanding of the scale values. By comparison, the variable-based model and independent samples *t*-test made few demands on the experimenter with regard to the meaning of the scale values, and they moreover required her to assume interval scale measurement and to assume that popularity itself is structured as a continuous quantity. No scientific evidence exists for either of these assumptions, and by thinking of her task as pattern identification the experimenter can avoid these assumptions while also pushing herself to think more deeply about what her numbers (i.e., the observations) actually mean.

**FIGURE 6:**
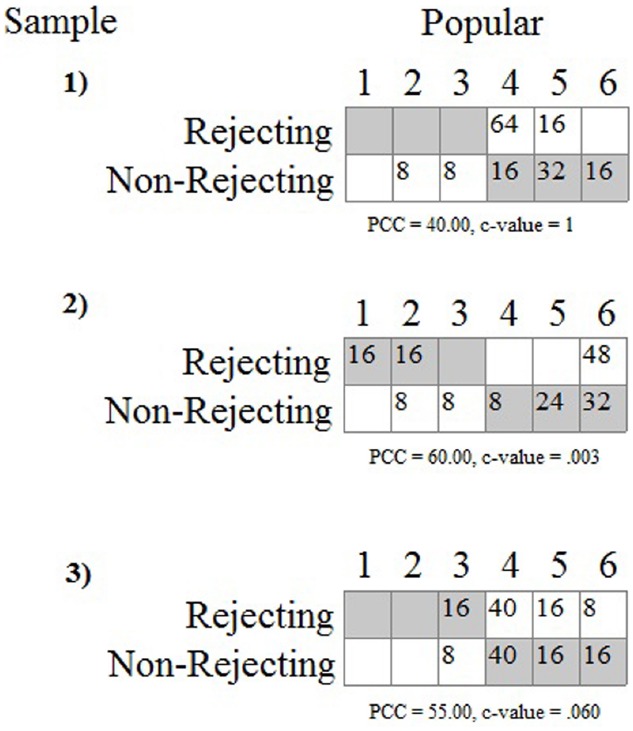
**Predicted pattern and actual observations for three samples from the rejection study.** Gray cells indicate predicted joint observations for group membership and popularity rating.

For the sake of demonstration, let us assume that the second pattern in Figure 5 is predicted by the integrated model. The actual observations from the three data sets can then be evaluated using the OOM software (Grice, 2011). The experimenter first sets up the two-dimensional array and defines the pattern. The frequencies are then computed and overlaid in the array, as shown in Figure 6. These are the primary results to be evaluated by the experimenter, and it can readily be seen that the observations from the first sample do not fit the pattern very well at all. None of the eighty participants in the rejected condition selected the 1, 2, or 3 values on the scale; and 16 participants in the non-rejecting condition selected these values, against expectation. Almost all of the 160 participants (90%) chose values of 4, 5, or 6. If these numerically high values are interpreted to represent the participant judging the counterpart as popular, and thus delivering a positive evaluation, then every person in the rejecting condition held a favorable attitude toward the counterpart.

Tallying all of the persons who were consistent with the predicted pattern yields what is known as the percent correct classification (PCC) index in the OOM software. The PCC index for the first sample was only 40%, as only 64 of the 160 joint group/rating observations matched expectation. The PCC index can range from 0 to 100 and is easily interpretable in light of Figure 6. A distribution-free randomization test can be conducted as an aid for interpreting the PCC index, the results of which are reported as a probability statistic known as the *c*-value (or *chance*-value). Relatively low values indicate the magnitude of the observed PCC index was not easily equaled or exceeded when computed from randomized pairings (1000 trials) of the group and popularity ratings for the 160 participants. For the first sample the *c*-value was 1.0 (possible range: 0–1), thus indicating that in every instance, the PCC index from randomized versions of the same data equaled or exceeded 40%. The observed PCC index was therefore not only low, but values at least that high were entirely ordinary as well.

The results in Figure 6 for the first sample are in direct contradiction to any reasonable expectation based on the integrated model. Yet, recall from Table 1 above the outcome from the *t*-test was statistically significant and interpreted as offering support for the variable-based model because the average rating for the rejecting group was lower than the average for the non-rejecting group. The place on the scale this difference occurred did not matter: the difference between 1 and 2 has the same meaning as a difference between 3 and 4 in the *t*-test analysis. With the OOM analyses, by contrast, the scale values had to be taken seriously when defining the expected pattern and interpreting the results.

The second data set also reveals striking results that were masked by the traditional statistics; specifically, as can be seen in Figure 6, 48 individuals (60%) in the rejecting condition rated the counterpart as a six on the 6-point scale. Again, these observations make no sense in light of the integrated model. The data for the rejecting condition are moreover split between the ends of the scale as the remaining 32 individuals selected 1’s or 2’s. Given this odd pattern the PCC index, which equals 60% and was unusual compared to randomized versions of the observations (*c*-value = 0.003, 1000 trials), is to be interpreted cautiously or even ignored. More specific analyses must also be conducted in this case, treating the two groups separately. The PCC index for the rejecting participants, treated separately, was only 40% (*c*-value = 0.98), while the PCC index for the non-rejected participants was impressively high (80%, *c*-value < 0.001). Expectations were therefore largely met for the non-rejected participants but not for the rejected participants.

Figure 6 shows the results for the third data set to be entirely unimpressive, even though the *t*-test was again statistically significant. As can be seen, a majority of the participants in the rejecting condition again chose 4, 5, or 6 from the rating scale. Equal numbers of participants in the non-rejecting condition chose 4’s and 5’s, and not a single individual from either group chose 1 or 2. The differences between the groups occurred only for values of 3 and 6, with more participants in the rejecting condition choosing 3’s and more participants in the non-rejecting condition choosing 6’s. The PCC index (55%) indicated that barely over half of the students were classified correctly which was even less impressive than the value obtained for the second data set, even though it was also unusual based on the randomization test (*c*-value = 0.06). Again, the results shown in Figure 6 are primary, and as a general rule in OOM PCC indices and *c*-values should never be presented without clear visual displays of the data. Opposite of NHST, as well, the probability statistic (viz., the *c*-value) is the least important bit of information in the analysis, and in this particular set of analyses it may even be considered superfluous.

### Abductive, *post hoc*, Pattern Evaluation

The model in Figure 1 does not explicitly predict which values on the 6-point scale will be chosen by individuals in the two groups. Without such specificity in the model, the experimenter must approach the three data sets in a manner consistent with inductive and abductive reasoning. In the parlance of modern research design and statistical analysis, she must examine the data *post hoc* for robust and meaningful patterns. She can do so using the OOM software and what is known as binary Procrustes rotation, which is a procedure that seeks to rotate one set of observations into conformity with a second set of observations (Grice, 2011).

**FIGURE 7:**
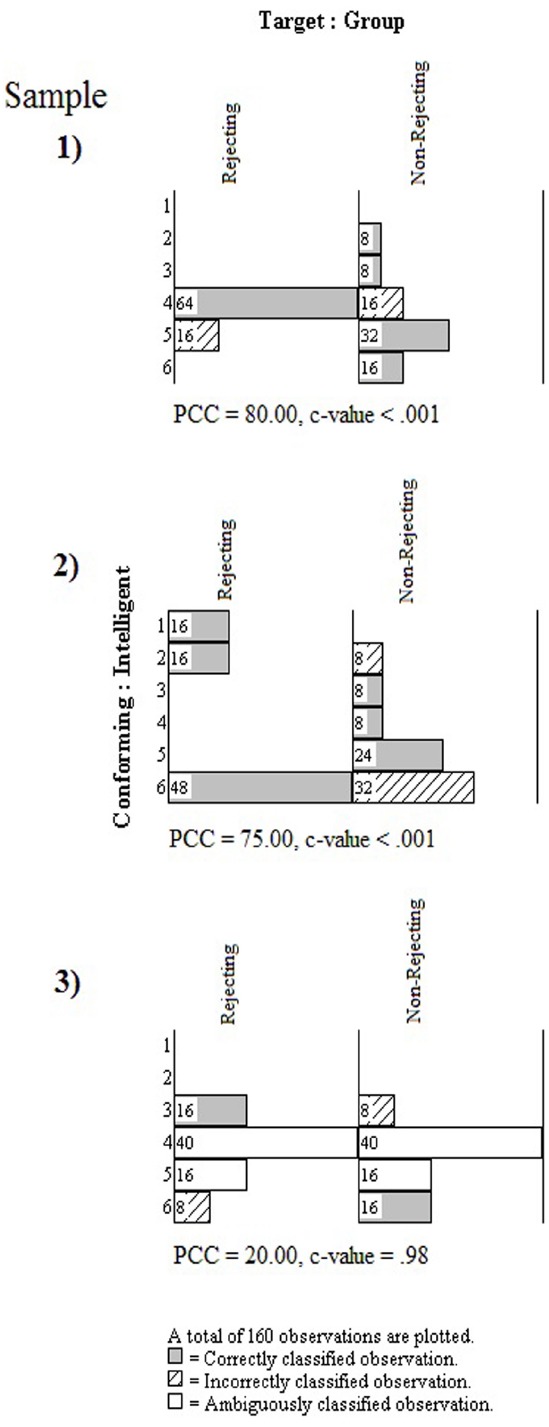
**Multigrams for three samples from the rejection study**.

Results for the three data sets, displayed as multigrams, are shown in Figure 7. As can be seen for the first sample, the multigram is comprised of two aligned histograms for the rejecting and non-rejecting groups. The bars in the multigram are shaded or filled on the basis of the Procrustes rotation. A shaded bar indicates those observations that are considered correctly classified by the algorithm, while a bar filled with diagonal lines indicates those observations incorrectly classified. It is important to keep in mind that the analysis is entirely *post hoc*. The observations are classified as correct or incorrect by the rotation algorithm on the basis of the patterns of frequencies considered both between groups and across the six scale values. The experimenter in no way determines how the observations are expected to be patterned or considered as accurately or inaccurately classified. She must instead examine the pattern in the multigram and attempt to draw an inductive generalization and an abductive explanation.

The multigram for the first sample shows a convincing pattern with regard to the PCC index (80%). As can be seen in Figure 7, the largest bars in the multigram are shaded to indicate the correctly classified observations. The *c*-value from the randomization test is also impressively low. Not one time in 1000 trials did randomized versions of the actual observations yield a PCC index of 80% or more. The pattern is thus unusual, leading the experimenter to inductively reason that some phenomenon has potentially been detected. At the same time, the experimenter is confronted with the pattern in Figure 7 and must work abductively to explain it. As can be seen, rejected participants were classified correctly only if they chose 4 on the 6-point scale. Non-rejected participants were classified correctly if they chose 2, 3, 5, and 6. How does the model in Figure 1 comport with such a pattern? It is difficult to reconcile this observed pattern with the structures and processes in the integrated model, but the experimenter must try to do so. Alternatively, she can seek to modify the integrated model to explain the pattern. In either case, she is reasoning abductively as she ultimately seeks to make an inference to best explanation of the phenomenon she’s detected through her study.

If the experimenter has all three data sets to work with, however, it is clear that a single pattern has not emerged. The multigram for the second sample in Figure 7 shows that, contrary to the first sample, no participants in the rejecting group selected 4 from the scale; they selected only 1’s, 2’s, or 6’s, with a majority choosing 6’s. A majority of participants in the rejected group of the third sample chose 4’s, but an equal number of participants chose 3’s, 5’s, or 6’s. The distributions of observations across the six scale points for the non-rejected participants were, by comparison, more similar across all three samples, although the modes were different for each. The PCC index for the second sample was high (75%) and unusual (*c*-value < 0.001), whereas the PCC index for the third sample was low (20%) and easily equaled or exceeded by randomized versions of the data (*c*-value = 0.98). The pattern for the third sample also shows ambiguous classifications, indicating that the algorithm could not clearly distinguish between values of 4 and 5 for the rejected and non-rejected participants. Given the low PCC index and high *c*-value for this sample, the experimenter would interpret these results as not supporting the integrated model, and the data offer no clear pattern from which to generalize or alter the model. The relatively impressive individual results for the second sample would warrant further abductive attention. Considering all three sets of results together with their remarkably different patterns, however, may instead decide to conclude that no phenomenon has been reliably detected.

## Discussion

Speelman and McGann (2013) report a number of assumptions about the mean held by modern psychologists. In light of the history, models, methodology and data analytic techniques examined in this paper, perhaps the most troubling assumption is that “any inability to use the mean as a reliable measure of a stable characteristic is a product of weakness in methodology or calculation” (p. 3). This assumption is disturbing for two reasons. First, how is it possible that one, simple statistic can be given so much power in the vast domain of scientific inquiry? Surely the spectacular advances in the fields of biology, chemistry, physics, and medicine, with all of their methodological rigor, have not depended on the lowly mean. The curious elevation of the mean in psychology as an indicator of rigor or as some type of “error free value”—or worse, “ideal person”—is the epitome of what Lamiell (2013) termed “statisticism.” Philosophically, it is the error of placing methods before metaphysics; in other words, allowing methods of data collection and analysis to determine how one builds a model of nature. The practical result of such a limiting attitude is a guaranteed restriction in the advancement of psychological science.

Second, the idolatry of the mean is disturbing because it reveals that psychologists are operating under a quantitative imperative (Michell, 1999, 2008). What is popularity? What is rejection? What is the emotion of anger? Under the quantitative imperative the answer to each of these questions must in some way invoke the notion of continuous quantity. In other words, each of these qualities of human experience are presumed to exist in such a way as to be measurable as continuous quantities. In the parlance of Steven’s, (1946) four scales of measurement, popularity, rejection, and emotion must be measured as interval or ratio scales, for it is only with these types of scales that the computation of a mean is appropriate. Unfortunately, there is no evidence to date that qualities such as intelligence, depression, and personality traits (let alone popularity, rejection, or anger) are structured as continuous quantities, and therefore measurable as such. As stated by Michell (2011), “There is no evidence that the attributes that psychometricians aspire to measure (such as abilities, attitudes and personality traits) are quantitative” (p. 245). This is again an instance of putting methods ahead of metaphysics; that is, of presuming psychological qualities to be measurable as continuities without substantiating this claim and seriously considering the possibility that such qualities may be structured differently.

One need only examine the periodic table of the elements or the biochemical pathways of a eukaryotic cell to understand that the scientific study of nature is not restricted to interval and ratio scaled measurement and parametric statistics. The arguments, models, and methods, presented in this paper hopefully elucidate why psychologists should feel confident in venturing beyond the world of means, variances, and covariances without fearing a loss of scientific rigor. Placing the integrated model in Figure 1 side-by-side with the variable-based model in Figure 2 should be sufficient to convince the reader that theoretical rigor is in no way tied to an aggregate statistic of any kind. Many of the components in Figure 1 (e.g., all of the acts of predication and most acts of judgment) are not even quantitative in nature, thus precluding the computation of a mean and variance. An integrated model like the one in this paper clearly requires a great deal more thought and effort to construct, validate, and defend than a variable-based model (see also Grice, 2011, 2014; Grice et al., 2012). Indeed, the reader is invited to sketch an integrated model for his or her most recent study, posited psychological process, or favorite theory. The task will no doubt prove challenging, but it will finally heed Meehl’s (1978) call for more serious theorizing and bolder predictions in psychology. Not by coincidence, in the same paper Meehl argued that the over-reliance on null hypothesis significance testing was preventing scientific progress in psychology,

“I believe that the almost universal reliance on merely refuting the null hypothesis as the standard method for corroborating substantive theories in the soft areas is a terrible mistake, is basically unsound, poor scientific strategy, and one of the worst things that ever happened in the history of psychology” (p. 817).

The shift to iconic modeling is also a step toward the types of inferences psychologists truly wish to make from their research: abductive inference, inference to best explanation, and inductive generalization. Variable-based models are meant to show associations between variables and are poor tools for explaining the complex structures and processes of nature. The mean does not provide information about “people in general” and in fact likely describes no one in particular (Lamiell, 2013). Variable-based models and their accompanying aggregate-based analyses are therefore not up to the task of delivering these inferences. When psychologists employ such methods and tie them to null hypothesis significance testing (traditional *p*-values), they are limited to drawing inferences about population parameters…regardless of whether or not they are cognizant of this fact. Using Haig’s (2005) ATOM, these inferences may be of value insofar as they are seen as equivalent to phenomenon detection. The Flynn Effect, for instance, is the phenomenon of increased scores on intelligence tests over the past 30 years or so “detected” using aggregate statistics (Haig, 2014). The explanation of this phenomenon, however, will require a great deal more work and the construction of an integrated model that details the structures and processes underlying the Flynn Effect.

Going beyond the world of variable-based modeling and the computation of means, variances, and other parametric statistics is not necessarily a leap into the world of Bayesian statistics or non-parametric analyses; rather, the move is from estimating parameters in the context of sampling variability (as with an independent samples *t*-test) to the analysis of patterns in the context of explanatory models. Thorngate (1986) wrote plainly, “The essence of science is the detection and explanation of patterns” (p. 71), and he wrote this statement in a chapter for a book titled *The Individual Subject and Scientific Psychology* (Valsiner, 1986). Countless students have entered psychology expecting to study the lives of individuals only to learn that their task is instead to study variables, aggregates and some non-existent “average person.” When collecting and analyzing data they learn that the odd person is a statistical nuisance or outlier who must be sacrificed to the mean or some statistical assumption (e.g., homogeneity). After all, the primary goal is to estimate population parameters, and one cannot let an influential case or two unduly influence the estimates. In contrast, the methods shown in this paper represent a return to the person or persons in psychology. Because these methods are primarily visual in nature and do not rely on the computation of parametric statistics, outliers or assumptions of normality, homogeneity, etc., are never a concern. The Percent Correct Classification index is a simple frequency, and therefore an aggregate statistic, but it is always interpreted in light of a pattern (e.g., the *a priori* pattern or a multigram) and the complete set of observations. The simple “eye test” or more severe “interocular traumatic test” (Edwards et al., 1963) is taken seriously in OOM as there simply is no substitute for examining the data, particularly in light of an integrated model.

The final move, then, is from variable-based models to persons. The example study above employed a between-group design, and only two pertinent observations were made for each participant. A more intensive study of the individual is possible, however, using similar methods to conceptualize and analyze multiple observations made for each person. Cohn et al. (2014) for example, collected daily diary ratings from 54 women who had been raped. Ratings of post-traumatic stress disorder (PTSD) symptoms, drinking behavior, emotional states, and many other attributes, attitudes or behaviors were collected for 14 consecutive days. Using the OOM software in a novel analysis of the data, Grice et al. (in press) were able to examine a mediation model (PTSD → Negative Affect → Alcohol Consumption) at the level of the individual women. Unlike the aggregate results obtained from a variable-based Hierarchical Linear Model, the OOM analyses identified the individual women whose observations revealed a causal connection for each link in the model. In the world of clinical intervention where individuals—not means—are treated, such techniques are tantamount (Mumma, 2004; Haynes et al., 2009). Additional examples of person-centered studies using OOM have been published (e.g., Brown and Grice, 2012; Craig et al., 2012; Abramson et al., 2015), and methods of data analysis which permit a dynamic study of individuals have also been developed (e.g., see Valsiner et al., 2014). The time is therefore ripe for psychologists to return to a study of the person as an integrated, individual whole.

### Conflict of Interest Statement

The author declares that the research was conducted in the absence of any commercial or financial relationships that could be construed as a potential conflict of interest.
